# Limitations of fasting indices in the measurement of insulin sensitivity in Afro-Caribbean adults

**DOI:** 10.1186/1756-0500-7-98

**Published:** 2014-02-20

**Authors:** Debbie S Thompson, Michael S Boyne, Clive Osmond, Trevor S Ferguson, Marshall K Tulloch-Reid, Rainford J Wilks, Alan T Barnett, Terrence E Forrester

**Affiliations:** 1 Tropical Medicine Research Institute, The University of the West Indies, Mona, Kingston 7, Jamaica; 2 MRC Lifecourse Epidemiology Unit, Southampton General Hospital, Southampton, UK; 3 Department of Surgery, Radiology, Anesthesia and Intensive Care, The University of the West Indies, Mona, Kingston 7, Jamaica

**Keywords:** Insulin sensitivity, Whole body glucose disposal, Insulin sensitivity index, Homeostatic model assessment of insulin resistance

## Abstract

**Background:**

Insulin sensitivity can be estimated using glucose disposal rate (M) measured during a hyperinsulinemic euglycemic clamp (HEC) or insulin sensitivity index (S_I_) derived from a frequently sampled intravenous glucose tolerance test (FSIVGTT). The commonly used homeostatic model assessment of insulin resistance (HOMA-IR) which utilizes fasting glucose and insulin has been validated against M across several populations (r = 0.5-0.8). This study sought to validate HOMA-IR against S_I_ and M in an Afro-Caribbean population.

**Findings:**

Sixty participants completed a 180-minute FSIVGTT and another 50 completed a 150-minute hyperinsulinemic euglycemic clamp. In both groups, HOMA-IR was calculated and anthropometry and body composition using dual energy x-ray absorptiometry (DEXA) were measured.

FSIVGTT: The participants were 55% male, age 23.1 ± 0.05 years, BMI 24.8 ± 6.3 kg/m^2^ and % body fat 25.0 ± 15.2 (mean ± SD). HEC: The participants were 44% male, age 27.3 ± 8.1 years, BMI 23.6 ± 5.0 kg/m^2^ and % body fat 24.7 ± 14.2 (mean ± SD). While HOMA-IR, S_I_ and M correlated with waist, BMI and % body fat (*P-*values < 0.01) there were no significant correlations between HOMA-IR with either S_I_ or M-value (*P-*values > 0.2).

**Conclusions:**

In young Afro-Caribbean adults, HOMA-IR compared poorly with other measures of insulin sensitivity. It remains important to determine whether similar findings occur in a more insulin resistant population. However, HOMA-IR correlated with clinical measures of insulin sensitivity (i.e. adiposity), so it may still be useful in epidemiological studies.

## Findings

### Background

Reduced insulin sensitivity is a predictor of incident type 2 diabetes and atherosclerotic risk [1]. The hyperinsulinemic euglycemic clamp (HEC) is considered the gold standard *in vivo* measurement of insulin sensitivity by calculating whole body glucose disposal (M). As the clamp is carried out under hyperinsulinemic conditions, hepatic glucose output is generally suppressed [2] and thus M mostly represents peripheral insulin sensitivity. The frequently sampled intravenous glucose tolerance test (FSIVGTT) measures whole body insulin sensitivity through the calculation of the insulin sensitivity index (S_I_) [3]. As both these measures are complex, the homeostatic model assessment of insulin resistance (HOMA-IR) is one of the most frequently used methods of determining insulin resistance in large population based studies, since it is mathematically derived from single fasting glucose and insulin measurements.

HOMA-IR has been used to assess longitudinal changes in insulin resistance in persons with type 2 diabetes of various ethnic groups in order to examine the natural history of diabetes and to assess the effects of treatment [4]. It can also be utilized in non-diabetic populations as it allows *1*) comparisons of insulin sensitivity among persons with abnormal glucose tolerance and *2*) the longitudinal assessment of persons who later develop abnormal glucose tolerance [4]. HOMA-IR has been validated against the HEC in multiple studies across several populations (r = 0.5-0.8) [5]. Other indices that utilize fasting glucose and insulin data (e.g. quantitative insulin sensitivity check index, QUICKI) have questionable superiority to HOMA [6,7].

Afro-Caribbean adults have high rates of type 2 diabetes and the metabolic syndrome, but there has been no validation of these simple measures of insulin resistance in this population. Consequently, we compared a simple surrogate measure of insulin resistance/sensitivity, HOMA-IR to measures derived from HEC and FSIVGTT in 110 non-diabetic Afro-Caribbean adults.

### Methods

#### Study design

These data were derived from two observational studies that utilized either a clamp or a FSIVGTT in 110 non-diabetic Jamaican adults. Diabetes was excluded on the basis of the patient’s stated history, a normal fasting plasma glucose, as well as an oral glucose tolerance test. In both studies, we excluded persons who were pregnant, had renal impairment, used glucocorticoids or smoked. None of the participants were taking drugs that affect insulin action, viz. insulin sensitizers, hypolipidemic agents, oral contraceptives or anabolic steroids. We also measured anthropometry using a standardized protocol [8] and body fat using dual energy x-ray absorptiometry.

The Faculty of Medical Sciences/University Hospital of the West Indies Ethics Committee approved the protocols and all participants gave written informed consent.

#### Data collection

In the first study [9], we recruited 60 participants age 22–23 years old from the Jamaican Birth Cohort. A modified FSIVGTT was performed in the morning after a 10 hour fast. After securing intravenous access in the right antecubital vein, a 0.3 g/kg bolus of 50% dextrose was given over 1 minute at time zero. At 20 minutes, 0.03 IU insulin per kg body weight was injected into the right antecubital vein. Blood was collected at −10, −5, 0, 2, 3, 4, 5, 6, 8, 10 12, 14, 19, 22, 25, 30, 40, 50, 70, 100, 140 and 180 minutes to measure plasma glucose and insulin concentrations. Glucose was measured using glucose oxidase assay and plasma insulin measured with an enzyme-linked assay (Immulite®, DPC, LA, CA). The assay had an analytical sensitivity of 2 μIU/ml and the intra-assay coefficient of variation (CV) was < 8%. The MINMOD Millennium 6.02 software (MINMOD Inc., Pasadena, CA, USA) was used to calculate S_I_.

We performed 150-minute hyperinsulinemic euglycemic clamps in another study of 50 non-diabetic adults [10]. This included 40 survivors of childhood malnutrition and 10 age, sex and BMI-matched community controls who were never exposed to malnutrition. The studies were conducted in the morning after a 10 hour overnight fast. We inserted a retrograde intravenous cannula into a right dorsal metacarpal vein, and then the right hand was placed in a warm box maintained at 50°C to arterialize venous blood for intermittent sampling. Insulin was infused into a left antecubital vein at a rate of 1 mIU/kg/min and blood glucose was clamped at 5 mmol/l by means of a variable rate infusion of 20% dextrose water. During the procedure, plasma glucose concentrations were measured every 5 minutes from the right cannula with a glucose analyzer (YSI Instruments, Yellow Springs, OH). Additionally, blood was collected every 10 minutes in a fluoridinated tube to measure glucose and a heparinised tube to measure insulin levels. Glucose was measured in the laboratory using glucose oxidase and plasma insulin measured using an immunoassay technique (ALPCO, Salem, NH). The intra-assay coefficient of variation was 3.13%.

The whole-body glucose disposal rate (M) was calculated as the mean of the glucose infusion rate during steady state of the clamp as follows:

*M* = *GIR* − *SC*;   where GIR was the glucose infusion rate and SC the space correction.

SC (mg/kg/min) = (G_2_-G_1_) × 0.63.

The steady state was defined as a 30 minute period occurring after 120 minutes, during which the coefficients of variation for blood glucose and GIR were less than 5% [11].

For all 110 participants, HOMA-IR was calculated as follows [12]:

$$\mathrm{HOMA}-\mathrm{IR}=\frac{\mathrm{fasting}\;\mathrm{insulin}\;\left(\mathrm{uIU}/\mathrm{ml}\right)\;\times \;\mathrm{Fasting}\;\mathrm{glucose}\;\left(\frac{\mathrm{mmol}}{L}\right)}{22.5}$$

In our laboratory, the correlation between the insulin assays used in both studies was 0.9.

#### Statistics

Continuous variables are presented as means ± SDs and in highly skewed data we used medians with interquartile ranges. HOMA-IR, S_I_ and M were log transformed to a normal distribution before the use of parametric tests. Comparisons of means were assessed using 2 sample *t*-test. Partial correlation coefficients were used to explore the associations between HOMA-IR with M and S_I_, controlling for age and sex. Statistical analyses were performed using SPSS 16.0 (SPSS, Chicago, IL). *P*-values ≤ 0.05 were considered statistically significant.

### Results

The clinical characteristics of our study populations are shown in the Table 1. The two groups did not differ with respect to age or adiposity but the HEC group was more insulin resistant using HOMA-IR (p < 0.0001).

**Table 1:** Clinical and metabolic characteristics of Afro-Caribbean adults who had FSIVGTT or HEC

**Characteristics**	**FSIVGTT**		**HEC**	
	**Male (n = 33)**	**Female (n = 27)**	**Male (n = 22)**	**Female (n = 28)**
**Age (years)**	23.1 ± 0.5	23.1 ± 0.5	27.6 ± 9.1	27.0 ± 7.4
**Height (cm)**	177.0 ± 5.5	164.8 ± 7.2	171.7 ± 7.8	158.7 ± 7.1
**Weight (kg)**	73.4 ± 12.0	72.9 ± 25.3	69.0 ± 12.6	60.3 ± 16.2
**BMI (kg/m**^**2**^**)**	23.4 ± 3.4	26.6 ± 8.4	22.3 ± 3.8	23.8 ± 5.8
**Waist (cm)**	77.4 ± 8.1	81.4 ± 16.8	78.1 ± 9.9	75.7 ± 14.5
**Percent body fat (%)**	14.6 ± 8.8	37.7 ± 11.6	13.7 ± 8.7	33.3 ±11.4
**Fasting glucose (mmol/l) l/L**	5.1 ± 0.3	4.9 ± 0.4	4.1 ± 0.7	4.3 ± 0.5
**Fasting insulin (uIU/ml)**	2.0 (2.0, 2.0)	2.0 (2.0, 5.2)	10.4 (3.0, 17.6)	8.0 (4.5, 14.6)
**M (mg.kg**^**-1**^**.min**^**-1**^**)**	-	-	9.0 (5.9,12.1)	7.3 (5.2, 10.2)
**S**_**I **_**[min**^**-1**^**(μU/ml)]**	2.2 (1.3, 3.4)	1.9 (0.6, 2.8)	-	-
**HOMA-IR**	0.44 (0.42, 0.52)	0.44 (0.40, 1.65)	1.6 (0.7, 3.0)	1.6 (0.9, 2.5)

#### FSIVGTT

**Figure 1:**
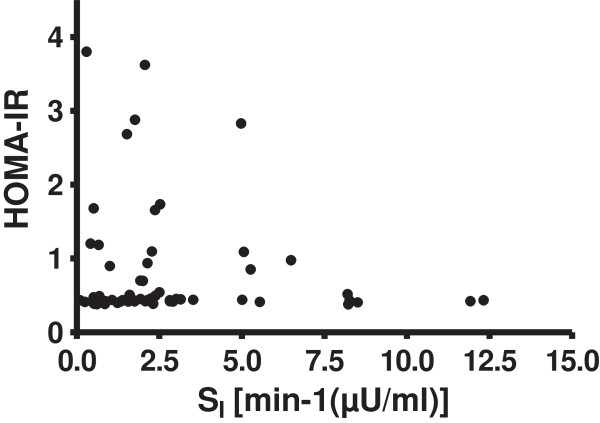
**Correlations between HOMA-IR and S**_**I **_**in non-diabetic Afro-Caribbean adults (r = −0.14, *P*** **= 0.19).**

The participants were 55% male, age 23.1 ± 0.1 years and BMI 24.8 ± 6.3 kg/m^2^. HOMA-IR did not correlate with S_I_ (Table 2, Figure 1) even after further adjustment for BMI (r = 0.16, *p* = 0.25). However, HOMA-IR had significant positive associations with weight, BMI, waist circumference and % body fat. S_I_ had negative associations with weight, BMI, waist and % body fat (Table 2).

**Table 2:** Correlations (r) of clinical and metabolic characteristics with measures of insulin sensitivity in Afro-Caribbean adults

**Measurement**	**FSIVGTT (55% male) N = 60**		**HEC (44% male) N = 50**	
	**Correlations (r)**		**Correlations (r)**	
	**HOMA-IR**	**S**_**I**_	**HOMA-IR**	**M**
**Age (years)**	−0.08	0.05	−0.14	−0.18
**Height (cm)**	0.23	0.03	−0.11	−0.02
**Weight (kg)**	0.39^*^	−0.34^*^	0.01	−0.32*
**BMI (kg/m**^**2**^**)**	0.36^*^	−0.38^*^	0.08	−0.33*
**Waist (cm)**	0.39^*^	−0.39^*^	0.06	−0.50*
**Percent body fat (%)**	0.29^*^	−0.40^*^	0.10	−0.49*
**F glucose (mmol/l) l/L**	0.45^*^	0.08	0.07	−0.16
**F insulin (uIU/ml)**	0.99^*^	−0.02	0.99*	−0.16
**M (mg.kg**^**-1**^**.min**^**-1**^**)**	-	-	−0.18	1.00*
**S**_**I **_**[min**^**-1**^**(μU/ml)]**	−0.01	1.00^*^	-	-
**HOMA-IR**	1.00^*^	−0.01	1.00*	−0.18

Correlations are adjusted for age and sex; those that are statistically significant at the 5% level are shown with an asterisk.

#### HEC

**Figure 2:**
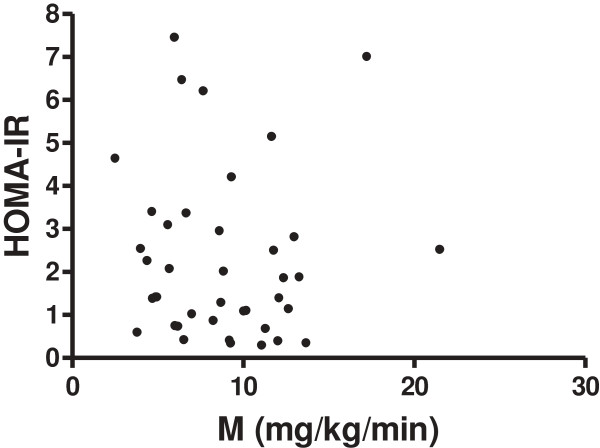
**Correlations between HOMA-IR and M in non-diabetic Afro-Caribbean adults (r = −0.01, *P*** **= 0.93).**

The participants were 44% male, age 27.3 ± 8.1 years and BMI 23.6 ± 5.0 kg/m^2^. There was no significant correlation between HOMA-IR and M (Table 2, Figure 2) even after adjusting for BMI (r = −0.17, *p* = 0.26). Additionally, no association between HOMA-IR and M was found in a subgroup of 7 individuals that were diagnosed for the first time with pre-diabetes during the study (r = −0.54; *P* = 0.22). M had significant negative associations with weight, BMI, waist and % body fat (*P*-values ≤ 0.04).

### Discussion

Fasting indices of insulin sensitivity compare poorly with estimates derived from HEC and FSIVGTT in our study population. While our findings contrast with data from other populations, they were consistent with the findings from studies in elderly diabetic Japanese and Greek women with polycystic ovary syndrome [13]. Pisprasert et al. concluded more recently that insulin sensitivity indices based on fasting glucose and insulin levels should be used cautiously as measures of peripheral insulin sensitivity when comparing mixed gender and mixed race populations [14]. Although our sample size was comparatively small, other studies that validated dynamic measures and HOMA-IR typically used 25–60 participants which is similar in size to our study [12].

There may be several factors that explain this apparent inconsistency. First, African-Americans secrete more insulin at any given level of insulin sensitivity [15] and their rates of insulin clearance may be lower than Caucasians [16]. Therefore in this population, fasting indices may overestimate insulin resistance. This would also produce stronger correlations in populations that are more insulin resistant and/or diabetic as opposed to our participants who were more insulin sensitive. While early beta cell failure could also alter fasting indices, this is unlikely to be the factor as African Americans have greater beta cell function compared to Caucasians [17].

Additionally, insulin resistance is a heterogeneous entity, that is, it impairs glucose uptake in several tissues especially muscle and adipose tissues, as well as it affects hepatic glucose output. Individuals may have varying phenotypes of insulin resistance and, therefore, the calculated indices may not completely reflect the *in vivo* milieu [13]. Consequently, one possible explanation for the lack of association in our population is a difference in impaired peripheral insulin action compared to hepatic insulin action. HOMA-IR and QUICKI are based on the feedback loop of glucose and insulin in the post-absorptive state and thus are more representative of hepatic insulin resistance and hepatic glucose output. Interestingly, in pre-pubertal and early pubertal adolescents, HOMA and fasting insulin were shown to reflect total body insulin sensitivity and hepatic insulin resistance, but not peripheral insulin sensitivity [18]. This is contrary to hyperinsulinemic clamps performed at steady-state serum insulin levels that suppress hepatic glucose production and directly reflect glucose disposal predominantly into skeletal muscle. On the other hand, the FSIVGTT combines the effects of insulin to promote glucose disposal in skeletal muscle and suppress hepatic glucose production [19]. As a result, fasting indices may correlate poorly with S_I_ and M in persons who mostly have peripheral insulin resistance. Afro-Americans have significant hepatic insulin resistance compared to peripheral insulin sensitivity [20]. This is so despite African Americans having less hepatic fat compared with Hispanics and Caucasians [20]. Whole body insulin resistance may therefore equate to a different, but overlapping set of metabolic derangements and may reflect tissue-specific differences in insulin signaling [20].

Another factor that may have influenced our findings is our relatively lean population. Fasting indices have been shown to be less accurate in subjects with normal or near normal weight [21]. The degree of obesity modifies the relationships among insulin resistance, insulin secretion and insulin catabolism such that plasma glucose and insulin concentrations are better able to delineate differences in more obese individuals [21]. Of note, however, was the observation that HOMA-IR did correlate with clinical measures of insulin resistance (i.e. adiposity) similar to S_I_ and M.

### Conclusion

In this first study exclusively investigating young, Afro-Caribbean adults, we showed that fasting indices are not equivalent to indices derived from the FSIVGTT or HEC. Our findings, however, may have been influenced by the fact that this population was lean and relatively insulin sensitive. Fasting indices may therefore be less reliable at predicting insulin sensitivity in this group, and, by extension, in studies involving multiple ethnic groups. In spite of this, fasting indices may still prove to be useful as a ranking tool in epidemiological studies in this population.

## Abbreviations

HEC: Hyperinsulinemic euglycemic clamp; SI: Insulin sensitivity index; FSIVGTT: Frequently sampled intravenous glucose tolerance test; HOMA-IR: Homeostatic model assessment of insulin resistance; M: Glucose disposal rate; DEXA: Dual energy x-ray absorptiometry; QUICKI: Quantitative insulin sensitivity check index.

## Competing interests

The authors declare that they have no competing interests.

## Authors’ contributions

DT collected the data and wrote the first draft of the manuscript. MSB contributed to the study design, data collection and interpretation of the data and reviewed the manuscript. CO contributed to the data analysis and the writing of the manuscript. TSF, MKTR and RJW contributed to the interpretation of the data, writing and review of the manuscript. AB contributed to data collection and the writing of the manuscript. TEF contributed to the study design, data collection, interpretation of the data, and writing of the manuscript. All authors read and approved the final manuscript.
